# Leptin dysfunction and the risk of dyslipidemia and metabolic syndrome in Romanian HIV-infected patients undergoing antiretroviral therapy

**DOI:** 10.1186/1471-2334-14-S4-O12

**Published:** 2014-05-29

**Authors:** Cătălin Tilişcan, Raluca Mihăilescu, Daniela Munteanu, Victoria Aramă, Ana Maria Tudor, Cristina Popescu, Adriana Hristea, Anca Ruxandra Negru, Roxana Petre, Iulia Niculescu, Mihai Lazăr, Daniela Adriana Ion, Adrian Streinu-Cercel, Sorin Ștefan Aramă

**Affiliations:** 1 National Institute for Infectious Diseases “Prof. Dr. Matei Balş”, Bucharest, Romania; 2 Carol Davila University of Medicine and Pharmacy, Bucharest, Romania

## 

Leptin is a hormone secreted by the adipose tissue that may be associated in the general population with components of the metabolic syndrome (MS). Our objective was to test the association between dyslipidemia, MS presence and circulating leptin dysfunction in a cohort of HIV-infected non-diabetic patients undergoing combinant antiretroviral therapy (cART).

We included HIV-infected non-diabetic consecutive patients undergoing cART, admitted to the National Institute for Infectious Diseases “Prof. Dr. Matei Balş”, between 2008-2011. The diagnosis of MS was made using the International Diabetes and American Heart Association harmonized criteria from 2009. Circulating levels of leptin (BioSource EASIA) were measured.

We enrolled 95 patients: 53 (55.8%) males (mean age=33.1±13.4 years) and 42 (44.2%) females (mean age=30.5±13.6 years). Most patients (72.5%) had undetectable HIV viral load; median CD4 count was 493.5 (IQR=422)/cmm. The median time from HIV diagnosis was 60 (IQR=73) months. The median time on cART was 58.5 (IQR=70) months, 53.8% of patients had experienced more than one cART regimen.

The prevalence of MS was 17.1%. Elevated blood pressure, elevated waist circumference and abnormal fasting glucose prevalences were 30.3%, 17.1% and 6.5%, respectively.

Median serum leptin was 1.89 (IQR=3.57) ng/mL. Circulating leptin dysfunction was present in almost half of patients, hypoleptinemia being more frequent (42.%) than hyperleptinemia (8.5%). Hypoleptinemia was more frequent in men (62.3%) comparative to women (17.1%), p=0.000.

The prevalence of MS in patients with hypoleptinemia was 25.8% vs 10.8% in persons with normal leptin values (p=0.261). Hypoleptinemia was associated with elevated waist circumference (p=0.004) and abnormal fasting glucose (p=0.05) in women. More than half (65.6%) of men with hypoleptinemia had reduced HDL-cholesterol levels vs 29.4% in men with normal levels of leptin. As expected, hyperleptinemia was associated with the increase of body mass index, both in men (p=0.000) and women (p=0.05).

In our cohort of young cART multiexperienced HIV patients leptin dysfunction was not significantly associated with MS presence. Leptin was correlated with several MS components (HDL-dyslipidemia, elevated circumference, abnormal fasting glucose) with significant gender differences, that suggests that leptin may play different roles in the regulation of glucose and lipid metabolism according to the sex.

